# Improving the implementation of tailored expectant management in subfertile couples: protocol for a cluster randomized trial

**DOI:** 10.1186/1748-5908-8-53

**Published:** 2013-05-20

**Authors:** Noortje M van den Boogaard, Fleur AM Kersten, Mariëtte Goddijn, Patrick MM Bossuyt, Fulco van der Veen, Peter GA Hompes, Rosella PMG Hermens, Didi DM Braat, Ben Willem J Mol, Willianne LDM Nelen

**Affiliations:** 1 Department of Obstetrics and Gynaecology, Academic Medical Centre, University of Amsterdam, P.O. Box 22660, Amsterdam DD 1100, The Netherlands; 2 Department of Obstetrics and Gynaecology, Radboud University Nijmegen Medical Centre, P.O. Box 9101, Nijmegen HB, 6500, The Netherlands; 3 Academic Medical Centre of the University of Amsterdam, Meibergdreef, 91105 AZ, Amsterdam, The Netherlands; 4 Department of Obstetrics and Gynaecology, Free University Medical Centre, PO Box 7057, 1007MB, Amsterdam, The Netherlands; 5 Scientific Institute for Quality of Healthcare (IQ healthcare), Radboud University Nijmegen Medical Centre, P. O. Box 9101, Nijmegen HB 6500, The Netherlands

## Abstract

**Background:**

Prognostic models in reproductive medicine can help to identify subfertile couples who would benefit from fertility treatment. Expectant management in couples with a good chance of natural conception, *i.e.*, tailored expectant management (TEM), prevents unnecessary treatment and is therefore recommended in international fertility guidelines. However, current implementation is not optimal, leaving room for improvement. Based on barriers and facilitators for TEM that were recently identified among professionals and subfertile couples, we have developed a multifaceted implementation strategy. The goal of this study is to assess the effects of this implementation strategy on the guideline adherence on TEM.

**Methods/design:**

In a cluster randomized trial, 25 clinics and their allied practitioners units will be randomized between the multifaceted implementation strategy and care as usual. Randomization will be stratified for *in vitro* fertilization (IVF) facilities (full licensed, intermediate/no IVF facilities). The effect of the implementation strategy, *i.e.*, the percentage guideline adherence on TEM, will be evaluated by pre- and post-randomization data collection. Furthermore, there will be a process and cost evaluation of the strategy. The implementation strategy will focus on subfertile couples and their care providers *i.e.*, general practitioners (GPs), fertility doctors, and gynecologists. The implementation strategy addresses three levels: patient level: education materials in the form of a patient information leaflet and a website; professional level: audit and feedback, educational outreach visit, communication training, and access to a digital version of the prognostic model of Hunault on a website; organizational level: providing a protocol based on the guideline. The primary outcome will be the percentage guideline adherence on TEM. Additional outcome measures will be treatment-, patient-, and process-related outcome measures.

**Discussion:**

This study will provide evidence about the effectiveness and costs of a multifaceted implementation strategy to improve guideline adherence on TEM.

**Trial registration:**

http://www.trialregister.nlNTR3405. This study is sponsored by ZonMW.

## Background

Subfertility is defined as the absence of conception after one year of unprotected intercourse [1]. It affects approximately 9% of all couples of reproductive age [2,3]. In approximately 50% of the couples, no major cause is found after the basic fertility work-up [4]. In those couples, the chance of natural conception can be calculated via validated prognostic models [5,6]. If the chance of natural conception within one year is good, meaning a probability of 30% or more, expectant management for 6 to 12 months is equally effective as treatment [7]. Because this expectant management is restricted to couples with a good prognosis, we have called it tailored expectant management (TEM).

European Society of Reproductive Medicine (ESHRE) and National Institute of Health and Clinical Excellence (NICE) guidelines on the management of infertility both emphasize that couples should not be exposed to unnecessary risks or ineffective treatments and encourage that each subfertile couple should receive information about the estimate of their chance of natural conception [8,9]. In the Netherlands, the national network guideline on infertility for gynecologists and general practitioners (GPs) explicitly recommends the use of prognostic models and subsequent TEM for couples with unexplained or mild infertility [10]. However, at this moment, implementation of TEM is poor. A recent Dutch multicenter cohort study showed overtreatment in 36% of the couples, *i.e.*, 36% of the couples with a good prognosis eligible for TEM (>30% chance of natural conception in one year) already started medically assisted reproduction (MAR) [11].

This overtreatment in subfertile couples is worrisome for several reasons. First, fertility treatment still leads to a considerable number of multiple pregnancies, which are associated with a higher morbidity and mortality in both mothers and neonates [12]. Second, fertility treatment carries a significant physical and psychological burden and accompanying productivity loss [13-16]. Third, fertility treatment and its complications are expensive and put considerable financial strain on societies where MAR is reimbursed or on the subfertile couples in societies where MAR is not or only partially reimbursed [17].

The first step to improve guideline adherence on TEM and reduce overtreatment is to gain insight into barriers and facilitators for implementation of TEM and MAR reduction. Subsequently a tailored implementation strategy can be developed targeting obstacles to change, if necessary at different levels [18,19]. In a previous qualitative and quantitative study among patients and professionals, the main barriers among subfertile couples were lack of confidence in natural conception, perception that expectant management is a waste of time, inappropriate expectations prior to the first secondary care consultation, and an overestimation of the success rates of treatment. Both couples and professionals regarded the lack of patient information materials as an important barrier. Among the professionals, limited knowledge about prognostic models and limited communication skills to convince the couple, both leading to a decision in favor of treatment, were recognized as main barriers. Facilitators experienced by the professionals were better management of patients’ expectations, local consensus, and the presence of a local protocol and local fertility meetings [11,20].

A multifaceted implementation strategy to improve guideline adherence on TEM has now been developed based on these data. The aim of this study is to evaluate effectiveness and costs of this implementation strategy in a cluster randomized trial.

## Methods

### Setting

In the Netherlands, subfertile couples are usually referred by the GP to secondary care. GPs usually perform only limited basic fertility workup or no workup at all and they do not prescribe fertility drugs. Secondary and tertiary care is provided by three different types of fertility clinics based on the kind of treatment they offer. Initial fertility assessment, ovulation induction (OI), and intra-uterine insemination (IUI) are carried out in all Dutch clinics. In vitro fertilization (IVF) and intracytoplasmic sperm injection (ICSI) treatments are only carried out in intermediate and licensed fertility clinics. Every Dutch citizen has a basic insurance coverage, which fully reimburses all treatment cycles of OI and IUI, with or without controlled ovarian hyperstimulation, as well as a maximum of three IVF or ICSI cycles.

### Study design

We propose a cluster randomized trial in 25 clinics and their allied GP units with an effect, process, and economic evaluation alongside the trial.

### Randomization

The 25 participating clinics and their allied practitioners units will be randomized between the multifaceted implementation strategy and care as usual. Randomization will be stratified for IVF facilities (full licensed, intermediate/no IVF facilities) and will take place after all clinics have approval to participate. Randomization will be done by an independent physician and will be computer-generated. Results of the randomization will be personally communicated to all participating clinics.

### Effectiveness

For the effectiveness, a baseline measurement will be performed in all participating clinics, including guideline adherence on TEM, and a range of organizational, professional, and patient characteristics (see outcome measures). Following baseline measurement, the multifaceted implementation strategy will be applied in the intervention clinics. After ten to twelve months of intervention exposure, the after measurement will be performed again in all 25 participating clinics.

### Process evaluation

A process evaluation, according to Hulscher *et al.*[21], will be performed during and after the intervention to investigate the feasibility of the implementation strategy.

### Intervention

The multifaceted implementation strategy, based on a barrier analysis among professionals and patients, will focus on three different levels: patient, professional, and organizational level [20]. The three levels and all associated tools are successively described here.

### Patient level

We will develop patient educational materials in three different forms, a patient information leaflet, posters, and a website.

The patient information leaflet will provide general background information on the fertility work-up procedure, prognostic factors that influence the chance on spontaneous conception, (dis)advantages of expectant management, and (dis)advantages of fertility treatment. In every intervention clinic, posters with information on the prognostic model and expectant management will be placed in the waiting areas. In the leaflet and on the posters, patients can find a code which is needed to gain access to the website. There is a different code for each intervention clinic. The website will give more individualized information by access to a digital version of the prognostic model of Hunault [5]. Herewith, patients can calculate their chances of natural conception within one year and experience the influence of altering characteristics. It will also provide additional information on the basic fertility workup, the chance of natural conception versus the chance of conception after fertility treatment, (dis)advantages of expectant management, and (dis)advantages of fertility treatment. Furthermore, it advises patients what they can do to optimize their chances of spontaneous conception during the expectant management period, *e.g.*, information on intercourse timing and frequency, weight regulation, and lifestyle. This information will be in accordance with the information provided in the Dutch national network guideline on infertility [10].

This information material will be developed according to the International Patient Decision Aids Standards criteria for the dimensions ‘information’ and ‘probabilities’ [22], as well as according to the American Medical Association criteria [23].

### Professional level (*e.g.*, gynecologists (in training), fertility doctors, and GPs)

The strategy regarding the professionals contains audit and feedback, an education outreach visit, supportive consultation tools, and a video-based communication training.

The audit and feedback of the current care will consist of a feedback report based on the results of the baseline measurement. This feedback will report clinic’s guideline adherence on TEM in a twelve-month period prior to the randomization compared with the other participating clinics. It will give insight in how they are adhering to the guideline concerning the policy for couples with unexplained or mild infertility, *e.g.*, use of prognostic models and subsequent TEM in case the prognosis is good. Furthermore, the report will provide feedback on patient-related measures like general experiences with fertility care, specific experiences with the prognostic model and TEM, quality of life, and trust in their physician [ref].

In addition to this audit and feedback, an educational outreach visit will take place with fertility doctors and gynecologists (in training), in which background information about how and when to use the prognostic model of Hunault, and subsequent TEM will be given and in which the results of the baseline measurement and local barriers will be discussed. The result of this visit will be an individualized action plan per clinic.

The supportive consultation tools are developed containing a digital version of the prognostic model of Hunault on a website and we will provide professionals with a summary of the guideline on TEM in the form of a pocket card.

Finally, a video-based training strategy will be provided to improve the communication techniques to counsel the patients on their chance of spontaneous conception versus conception after treatment, the (dis)advantages of expectant management versus fertility treatment, and on the reason for TEM (*i.e.*, making clear it is not a waste of time). Consistent with functional models of medical communication described in the field [24], the LEAPS Framework, a pneumonic for Listen, Educate, Assess, Partner and Support will be used to develop the intervention [25].

### Organizational level (GP units and fertility clinics)

During the educational outreach visits an example of an up-to-date local protocol will be offered to the fertility clinics that do not already have an updated protocol available. This local protocol will be based on the Dutch network guideline on infertility, and it will focus on the initial fertility assessment (diagnostics), identification of patients with mild or unexplained infertility, the use of the prognostic model of Hunault, and TEM [10]. The clinics can adjust this protocol to their own lay out and they can distribute it either in the form of a hard copy or digital copy, depending on the preference of the professionals.

Furthermore, we will provide the GPs allied to the intervention clinics with feedback on their referral behavior, *e.g.*, were patients referred according to the guideline recommendations.

### Study population/participants

To include a representative patient group, we will select potential participating couples retrospectively in each clinic by means of the clinics’ financial registration database (Diagnosis Treatment Combination code). In this nationwide registration system, patients undergoing diagnostics or treatment for infertility are identified with a specific fertility code (F-code). For the baseline measurement, we will invite couples that were given the code for new fertility patients (F-11) between February 2011 to March 2012 to participate in this study. For the after measurement, we will invite the couples that were given the F-11 code during the ten- to twelve-month intervention period. We will invite couples to participate by giving their permission for a medical record study and filling out a questionnaire.

The couples are eligible to participate when they have been diagnosed with unexplained or mild infertility, have a good prognosis (>30%) according to Hunault’s prediction model, did not have previous fertility treatment, and the female age is between 18 and 38 years. Couples with bilateral tubal pathology, severe male factor, or anovulation are not eligible to participate.

### Sample size

The expected adherence to TEM in the control arm is estimated based on previous studies at 60% [11]. To increase this to 80% with an intra-class correlation (ICC) of 0.1, alpha at 5%, comparing two strategies, we estimate that with 25 clusters we would need a total sample size of 450 patients. This means we need to include 15 to 20 patients per clinic in the baseline as well as in the after measurement.

## Outcome measures

### Primary outcome effectiveness

The primary outcome measure of the proposed study for effectiveness will be the guideline adherence rate on TEM: the percentage of couples that are eligible for TEM (couples with mild or unexplained infertility with a prognosis of >30% of natural conception within one year) who actually agree upon the expectant management period of at least six months after the initial fertility assessment is concluded.

### Secondary outcomes effectiveness

1. Treatment-related measures: time to the start of fertility treatment and the number and types of fertility treatments that the couples received.

2. Treatment outcome-related measures: (ongoing) pregnancy rate, miscarriage, extra uterine gravidity, multiple pregnancy rate, and time to pregnancy.

3. Patient-related outcome measures : general experiences with fertility care such as information provision, respect for patients’ values and accessibility of care (to be measured with Patient Centeredness Questionnaire Infertility) [26], specific experiences with the prognostic model and TEM, quality of life (estimated by FertiQoL)[27], and trust in physician (measured by Wake Forest Trust Scale) [28].

4. Process related measures: percentage transition of patients to another fertility center.

5. Background characteristics that could influence guideline adherence (*e.g.*, age, referral status, type of infertility, duration and cause of infertility).

### Outcomes process evaluation

1. Actual ‘exposure’ of patients and physicians to the different elements of the implementation strategy.

2. How frequently the website has been visited by patients and physicians.

3. Experiences of patients and physicians with the different elements of the implementation strategy.

## Data collection

### Effectiveness

Data collection will be performed from medical records and a patient questionnaire.

Data abstraction from medical records will be performed using a standardized audit form. We will collect the background characteristics, treatment related measures, treatment outcome-related measures, and process-related measures.

The questionnaire will be divided into four parts. The first part consists of background questions (*e.g.*, highest educational level, country of birth). The second part regards the patients’ experience with the prognostic model and TEM as well as the patients’ trust in both the GP as well as the fertility doctor/gynecologist. The third part is the Patient-Centeredness Questionnaire-Infertility, a validated instrument measuring patient-centeredness of fertility care by asking about patients’ experiences with care. The last part is the FertiQol questionnaire, We will only use the Core module, which involves questions about mind-body, emotional, relational, and social aspects.

### Process evaluation

For the process evaluation, we will approach the local investigator during the intervention period to provide us with feedback about the implementation strategy. We will also keep track of how often the website is visited by logging data. At the end of the ten- to twelve-month intervention period, we will evaluate the strategy by means of a professional questionnaire and an addendum to the patients’ questionnaire in the after measurement.

### Data analysis

To analyze the effectiveness of the implementation strategy, descriptive statistics and multilevel analysis will be used. The statistical analysis will be performed using SPSS version 16.0 for Windows.

The main outcome, the difference in baseline and after-measurement scores in guideline adherence on TEM, between the intervention and control group will be analyzed with the chi-square test.

Descriptive analysis will be used to assess the difference in treatment-related, treatment outcome-related, and patient-related measurements between the intervention and control group. Furthermore, time to pregnancy and time to start fertility treatment will be analyzed using Kaplan Meier analysis with log-rank test. Univariate and subsequent multivariate logistic and Cox regression analyses will be used to analyze the relative contribution of the implementation strategy versus other predictive factors for guideline adherence on TEM.

### Economic evaluation

We plan an economic evaluation alongside the clinical trial to investigate the cost-effectiveness of the multifaceted implementation strategy to improve guideline adherence on TEM. This economic evaluation compares the multifaceted implementation strategy to usual care and is done from a societal perspective. A distinction will be made between costs of the development and introduction of the implementation strategy and the costs of maintaining the implementation strategy. The input of resources is assessed by collecting volumes of consumed resources (*e.g.*, medical interventions like number IUI and IVF cycles and treatment related outcomes like ovarian hyperstimulation syndrome and multiple pregnancies) and multiplied by reported or guideline prices according to Oostenbrink [29]. To assess non-medical and indirect costs, we will build on the data collection and cost calculation frameworks from previous cost studies on IUI and IVF [30,31]. The incremental costs, expressed as costs/percentage guideline adherence to TEM, are determined by the differences in resource consumption and adherence rates between the intervention group and the control group. Robustness of the results (costs and health outcomes) for various assumptions and parameters estimates will be explored in sensitivity analyses and visualized in incremental cost-effectiveness ratio graphs and cost-effectiveness acceptability curves.

### Trial status

We are currently performing the baseline measurement in all participating clinics.

## Discussion

This cluster RCT will compare a multifaceted implementation strategy to usual care on improving guideline adherence to TEM. If TEM is applied more frequently, it will reduce the number of performed IUI, IVF, and ICSI cycles, the incidence of treatment related complications (*e.g.*, ovarian hyperstimulation syndrome and multiple pregnancies), and we expect it to reduce the physical and psychological burden. As a consequence, the costs for fertility treatment will decrease.

Many different interventions are available to implement guidelines, either focusing on professionals, patients, teams or organizational factors, and with variable effects. A systematic review on interventions to improve guideline implementation showed that interventions tailored to prospectively identified barriers are more likely to improve professionals practice than only dissemination of guidelines [32]. The strategy that we developed is tailored to the recently identified barriers and facilitators for TEM, thus more likely to improve professionals practice, in this case, adherence to the guideline. Moreover, in general, combined interventions are believed to be more effective than single interventions [32]. Therefore, to increase the potential effectiveness of our implementation strategy, we developed a multifaceted intervention that targets the specific barriers for TEM at different levels.

For the specific interventions that will be used in the multifaceted implementation strategy, the review showed that audit and feedback and educational outreach visits can be effective (small to moderate) [32-34] and patient-directed interventions such as educational materials may result in moderate to large effects to increase adherence to recommended care [32]. Moreover, it has been proven that subfertile patients appreciate education and improved knowledge, and it has also been demonstrated to influence their healthcare decisions [35,36]. In a systematic review, only the effect of paper version materials was studied. However, because surveys have shown that online health information retrieval and eHealth activities are becoming increasingly common, especially within young and highly educated subfertile patients [37-40], we decided to offer the patient information materials in both paper and digital forms (*i.e.*, website and application). By doing so, and thus tailoring the patient-directed intervention to the infertility population, we hope to increase the effect of this intervention even more.

Aside from the multifaceted and barrier tailored aspects of the strategy, this study has several more strengths.

First, the number of participating clinics is a great strength of this study. One-quarter of all Dutch clinics from all over the country participate in this study, ensuring the representativeness of the Dutch infertility population as well as the professionals who provide fertility care.

Second, the process evaluation provides us with more information on the effectiveness and usefulness of the different interventions used in the strategy and not only of the multifaceted implementation strategy as a whole. We know that the multifaceted aspect of the intervention does not necessarily make the intervention more effective, therefore we need to assess the effectiveness of each individual intervention separately as well [36]. This is of great value for further implementation research and development of implementation strategies. Furthermore, if the multifaceted implementation strategy proves to be effective, it could also be generalized to improve implementation of other guidelines.

Third, the cost evaluation that will take place is very important from a societal aspect. Healthcare is becoming increasingly expensive, and cost reduction is a very important and common topic in most governments and healthcare institutes. This economic evaluation will provide further information on how we can reduce costs in healthcare by following the current and already existing guidelines for best practice and care.

A possible limitation of the study is the chance of contamination of the GPs between the intervention and control group. GPs can refer patients to more than one clinic; this makes it possible that a GP who is allied to an intervention clinic can also refer patients to a control clinic. However, we think that the occurrence of actual contamination will be very small because the participating clinics are very well spread over the country. In case contamination of GPs does occur, we expect the effect on the outcome to be very small or even undetectable because the multifaceted intervention strategy is mostly targeted at the secondary and tertiary care.

In summary, the main contribution of this study is that it seeks to identify the most effective strategy for implementing the guideline on TEM in subfertile couples. Ensuring the appropriate uptake of guideline recommendations by both professionals and patients will improve the care for these patients.

## Abbreviations

TEM: Tailored Expectant Management; ESHRE: European Society of Reproductive Medicine; NICE: National Institute of Health and Clinical Excellence; NVOG: Dutch Society for Obstetrics and Gynecology; MAR: Medically Assisted Reproduction.

## Competing interests

The authors declare that they have no competing interests.

## Authors’ contributions

BWJM, WLN, PGH, FvdV, DB, RH, FAMK, and NvdB were involved in conception and design of the study. NvdB, FAMK, and WLN drafted the first manuscript. All authors read and approved the final manuscript and are local investigators in the participating centers.
